# A system approach to improving maternal and child health care delivery in Kenya: innovations at the community and primary care facilities (a protocol)

**DOI:** 10.1186/s12978-017-0358-6

**Published:** 2017-08-29

**Authors:** Fabian Esamai, Mabel Nangami, John Tabu, Ann Mwangi, David Ayuku, Edwin Were

**Affiliations:** 1 Child Health and Paediatrics, Department of Child Health, Eldoret, Kenya; 2 Paediatrics, School of Medicine College of Health Sciences Moi University, Eldoret, Kenya; 3 Department of Health Management and Health Policy, School of Public Health, College of health Sciences, Moi University, P. O Box 4606 30100, Eldoret, Kenya; 4 Department of Disaster Risk Management, School of Public health, College of Health Sciences, Moi University, P. O Box 4606 30100, Eldoret, Kenya; 5 Department of Behavioural Sciences, School of Medicine College of Health Sciences, Moi University, P. O Box 4606 30100, Eldoret, Kenya; 6 Department of Reproductive Health, School of Medicine College of health Sciences Moi University, P. O Box 4606 30100, Eldoret, Kenya

**Keywords:** Health systems, Maternal, Neonatal, Enhanced health care, Find link treat and retain

## Abstract

**Background:**

Maternal, fetal and neonatal mortality are higher in low-income compared to high-income countries due to weak health systems including poor access and utilization of health services. Despite enormous recent improvements in maternal, neonatal and under 5 health indicators, more rapid progress is needed to meet the targets including the Development Goal 3(SDG). In Kenya these indicators are still high and comprehensive systems are needed to attain the targets of the SDG 3 by 2030. We describe the structure and methods of a study to assess the impact of an innovative system approach on maternal, neonatal and under-five children outcomes.

This will be implemented in two clusters in the Counties of Busia and Bungoma in Kenya. There will be 4 control clusters in Kakamega, UasinGishu, Trans Nzoia and Elgeyo Marakwet Counties in Kenya. The study population will be pregnant women, newborns and under-five children identified over the study period. The objective of the study is to improve access, utilization and quality of Maternal and Child Health care through a predesigned Enhanced Health Care System (EHC) that embodies six WHO pillars of the health system and community owned initiatives including Community Based Organisations and Income Generating Activities.

**Methods/Design:**

A five year quasi-experimental design will be used to compare the outcomes of the implementation of the EHC using the Find Link Treat and retain (FLTR) strategy in one cluster, community owned initiatives in one cluster and four control clusters at baseline and at the end of the study. A Baseline survey will be conducted in year one and an endline in the fifth year in which maternal, neonatal and underfive childhood outcomes will be compared.

**Discussion:**

The expected findings from the study include showing trends in improvement in the intervention clusters for morbidity, mortality, health service utilization and access indicators. Use of the health systems approach in health care provision is expected to provide a holistic improvement in the quality of care in the study populations in the intervention clusters that will lead to improved health indicators including morbidity and mortality. It is expected that the findings will inform health policy of the national and county governments in Kenya and worldwide.

## Plain English summary

Worldwide, the mortality rate for children under five dropped from 87 deaths per 1000 live births in 1990 to 51 per 1000 in 2011. Despite this enormous accomplishment, most countries in developing countries, including Kenya, did not meet the Millennium Development Goals 4, 5 and 6. Increasingly, child deaths are concentrated in the poorest regions and in the first month of life.

The goal of this project is to improve access and quality of Maternal and Child Health care through a predesigned Enhanced Health Care system and community owned initiatives including CBOs and IGUs.

The primary long term objective is to evaluate the impact of the implementation of an “Enhanced Health Care (EHC)” delivery care system through Find, Link, Treat and Retain (FLTR) strategy on antenatal care attendance, health facility deliveries, low birth weight and premature deliveries.

Community health workers supervised by trained skilled health workers to go house to house to find pregnant women and newborns (**Find**). They encourage prompt ANC/MCH clinic attendance and scheduled ANC/MCH clinic appointments using mobile phones at the time of their initial visit (**Link**). The ANC/MCH clinics in the facilities provide the complete EHC package to each subject (**Treat**). Subjects are tracked and defaulters followed up and their compliance ensured (**Retain).**

The expected outcomes include overall increase in antenatal care attendance, health facility deliveries, improved quality of care, a reduction in low birth weight and premature births, reduction in birth asphyxia, a reduction in neonatal sepsis and subsequent reduced maternal, infant and neonatal mortality.

## Background

Despite decades of independence, most countries in sub-Saharan Africa are characterized by underperforming health systems in terms of low funding, operational and management inefficiency, poor quality of health services, inequities in distribution of the health workforce, and low capacity for planning, budgeting, and governance [1]. Efforts to improve the performance of these health systems are complicated by contextual factors such as intricate political landscape, unstable economic environment and rapid population growth. Some of the consequences of the weak health system include rising disease burden including non-communicable diseases, new and re-emerging epidemics. Specifically, most countries in sub-Saharan Africa, with the exception of Rwanda, Ethiopia,Malawi, Cape Verde and Tanzaniadid not meet MDG goals 4 and 5 because of weak health systems [2]. Building health systems that are responsive to health needs of populationsrequires innovative application of technologies and human resource with the capacity to conduct research and use evidence to inform health policy making and practice.

Health systems research (HSR), is an evolving field of study that “is interdisciplinary in nature and aims to provide policy and practice relevant information that improves the health system as a whole by addressing the goals of equity, efficiency, effectiveness, and sustainability, ultimately leading to improved health status”[3]. The high maternal and child morbidity and mortality in Africa is testament to non-responsive health systems that perpetuate inequalities due to ineffective and unsustainable interventions [4–8].

Studies and reports from low and high income countries indicate that maternal, fetal and neonatal mortality are 10 to 100 fold higher in many low-income compared to high-income countries. The reasons for these discrepancies are many, but include the facts that many women and newborns receive little prenatal care and most deliver outside of health facilities that can provide lifesaving treatment for the mother, fetus and newborn [9].

In low-income settings, maternal mortality rates range from 150 to more than 1000 per 100,000 live births [2] while rates of stillbirth and neonatal mortality generally range from 20 to 40 per 1000 births [10, 11]. Intrapartum stillbirth, or those stillbirths that occur during labor and delivery, are an important indicator of the quality of obstetric care (Goldenberg 2008; [12, 13]). While in high-income countries, intrapartum stillbirths have nearly been eliminated, in low-resource settings, up to half of all stillbirths occur in the intrapartum period. Another measure of care is obstetric ‘near miss’ which has been defined by the World Health Organization (WHO) to comprise women who nearly died but survived a complication during pregnancy (Pattinson, 2011) [14]. Near misses are considered an important indicator of intra-partum maternal care and rates may be as high as 33 per 1000 in low-resource settings (Okong, 2007; [15, 16]). Because maternal mortality is relatively rare and therefore difficult to study, many have advocated using near miss cases as a surrogate measure of maternal mortality, although morbidity is an important outcome on its own.

It is widely accepted that 15% of all pregnancies have direct and indirect medical or obstetric complications that greatly increase the risk of mortality or severe morbidity for the mother and newborn. These complications include haemorrhage, hypertension, unsafe abortions and obstructed labour (UNICEF/UNFPA 2007). Indirect causes include malaria, anaemia, tuberculosis and HIV/AIDS. Universal access to high quality facility care substantially reduces mortality and morbidity from these conditions [17]. Poor quality of antenatal care result in failure to detect and manage these high risk pregnancies in low- and middle-income countries. This poor access to antenatal care and the obstetric complications contributes to high early neonatal deaths including still births. In high-income countries, access to prenatal care is nearly universal.

**Fig. 1:**
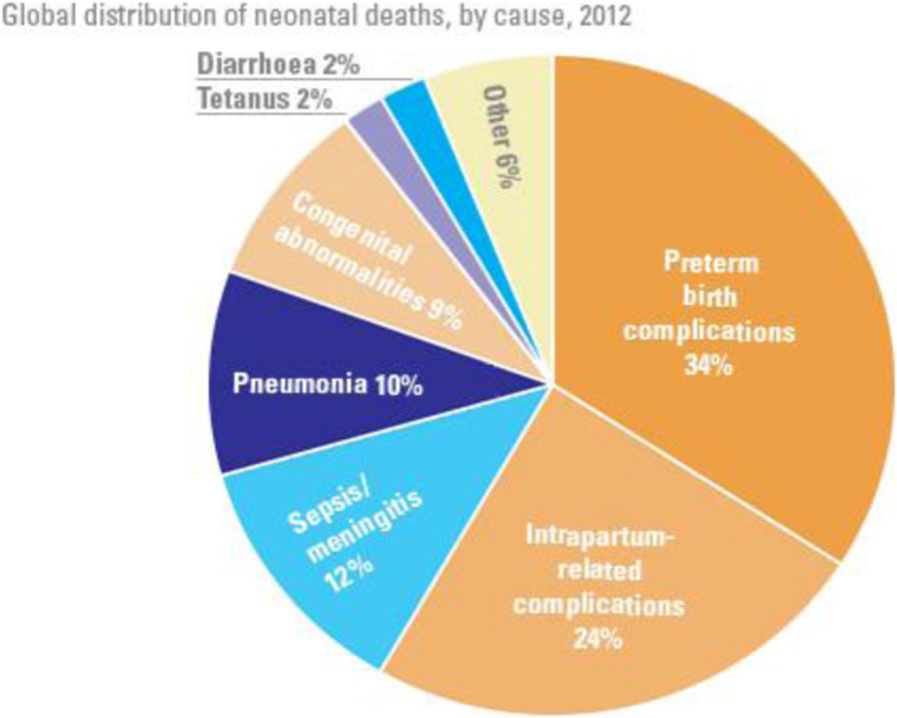
Causes of neonatal mortality worldwide, 2012. *Source:* [2]

In the neonatal period prematurity, infections and asphyxia account for about 80% of all deaths worldwide with most of these occurring in developing countries. The contributing factors to asphyxia, preterm births and neonatal sepsis are predominantly antenatal and intra-partum. Four in ten under-five deaths occur during the first month of life. Among children who survive beyond the first month, pneumonia, diarrhea and malaria are the leading killers with infectious diseases accounting for over two thirds of these deaths. Many of these deaths occur in children already weakened by under-nutrition. Worldwide, more than one-third of all under-five deaths are associated to malnutrition (UNICEF 2012, [18, 19]). Figure 1 summarizes the main causes of neonatal mortality, most of which are preventable.

**Fig. 2:**
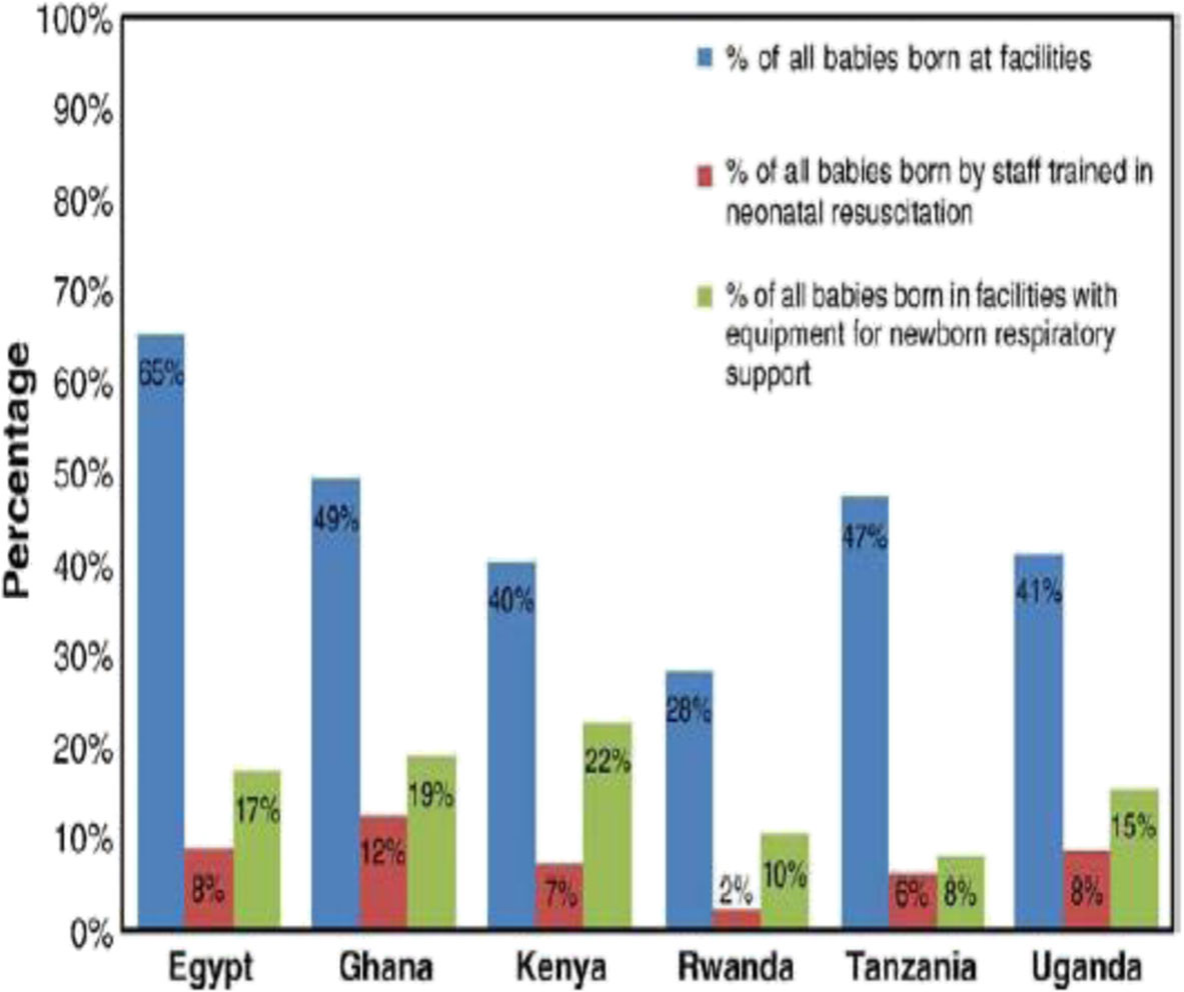
Status of where neonatal births and resuscitation occur in select African countries. *Source:*Wall SN, Lee ACC, Niermeyer S, et al. IJGO 2009; 107:S47

In developing countries, a significant proportion of deliveries occur outside health facilities and by unskilled birth attendants. In most health facilities, there is lack of equipment and drugs for basic obstetric, neonatal and child health care. In sub-Saharan Africa only between 20 and 70% of all births occur in health facilities, 5–15% of all newborns are resuscitated by a skilled health worker trained in neonatal resuscitation at time of birth and between 10 and 15% of these babies are born in facilities with resuscitation equipment. Facility delivery is known to be the single most effective intervention for preventing maternal and neonatal morbidity and mortality. However, this is only true if there is quality of care at the facility. In Kenya, about 60% of deliveries occur in health facilities and only 7% of newborns are resuscitated by skilled health workers trained on neonatal resuscitation and 22% are born in health facilities with resuscitation equipment (KDHS 2009, Goudar et al. 2011) [19, 20] as illustrated in Fig. 2.

### Problem statement

Maternal and neonatal mortality are unacceptably high in Sub-Saharan Africa compared to Western Europe and Northern America. This inequality is supported by the fact in Sub-Saharan Africa only three countries met their targets for MDG 4, 5 and 6(UN, 2013). The question is, why have a few countries succeeded yet they share similar socio-economic contexts with those that have not met their targets?. Since 2000, most countries have focused on interventions that aim to increase health facility delivery and neonatal and early childhood services – high impact interventions for MNCH. Evidence is emerging that during the same period health sector reforms in most Sub-Saharan countries, including Kenya, stalled and healthsystems have remained weak as characterized by inadequate funding (below the Abuja recommended 15% of the national budget), inefficient resource management and poor policy implementation, health workforce crisis, dilapidated infrastructure and weak health information systems [21].

One emerging challenge to achieving and sustaining desired targets for maternal and child health is neglect of the gap between the community and primary care health facilities, failure to espouse a systems/holistic approach as well as the continuum of the Three Delays Model [22]. In Kenya, the government acknowledged the importance of community-based interventions that stimulate demand for reproductive services by decentralizing the health planning and administrative functions to the district in 1984. However, there was no blue print or coordinated strategy to address the gap between the then level 1 and level 2 of the service delivery system. Although evidence is generally weak on maternal health indicators, information from KDHS (2003) showed that maternal, neonatal and child health indicators had either stalled or were worsening – primarily due to poor quality of care at health facility level (supply) and poor physical, financial and psychological access (demand) to reproductive health services. In 2006, the government formally recognized the importance of closing the gap (interface) between the community and front line facilities by launching the Community Health Strategy. Weak leadership as demonstrated by underfunding, uncoordinated and piecemeal implementation of the strategy has failed to appreciably improve the proportion of pregnant women who deliver at health facilities and by skilled attendants. Indeed some of the promising interventions have largely been small scale and unsustainable limiting the opportunities for replication and scale up.

#### Conceptual framework

**Fig. 3:**
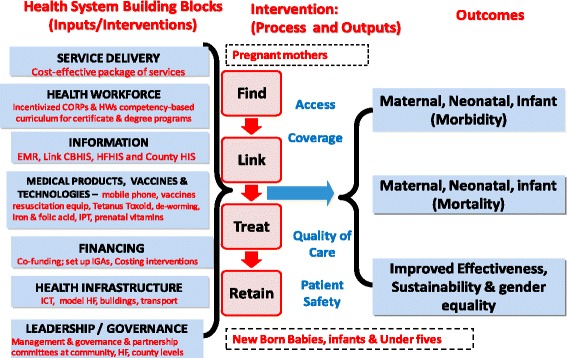
Conceptual framework: Linking Health System Inputs, Process, Outputs & Outcomes. Source: Adapted from [1]

The complexity of factors influencing maternal, fetal and neonatal mortality necessitate that the conceptual framework is guided by a holistic and systems perspective anchored on WHO’s framework for health systems which is also adopted in the Kenya’s current policy framework ([4, 23, 9, 11]). Figure 3 shows seven sub-systems, viewed as investment areas (inputs) that are linked to the interventions (process) leading to intermediate and long term outcomes.

The project aims to work closely within existing health systems to improve these intermediate outputs which will directly affect the scalability and sustainability of the study interventions. The long-term outcomes to be assessed at the end of the project include effectiveness and sustainability of the intervention in terms of reductions in maternal, fetal and neonatal morbidity and mortality as well as reductions ininequities such as gender.

#### Hypothesis

The implementation of an enhanced health care, shall lead to a significant increase in antenatal care attendance, increase in health facility deliveries and improved pregnancy outcomes. The intervention to be tested is an implementation process “*early identification of pregnantwomen and newborns at the community level, encouragement ofantenatal clinic/MCH attendance and assurance of actual delivery of ANC/MCH EHC interventions through follow up at community and facility levels by CHWs and SHWs respectively”.*

### The intervention: Enhanced health care package (EHC)

- Early identification and surveillance of the pregnant woman and newborn babies
- Recognition and management of pregnancy-related complications, particularly pre-eclampsia, haemorrhage, premature rupture of membranes and infection
- Recognition and treatment of underlying or concurrent illness in pregnancy – malaria, anaemia, infection among others
- Screening for conditions and diseases such as syphilis, HIV infection and hepatitis B
- Preventive measures through administration of tetanus toxoid, immunisation, de-worming, iron and folic acid supplementation, intermittent preventive treatment of malaria in pregnancy (IPT), prenatal vitamins provision and use of Long Lasting Insecticides Treated Nets (LLITNs)
- Developing a birth and emergency preparedness plan
- Immunization of all young infants and under-five children born during the study period including Vitamin A administration at 6 months of age and yearly
- Obtain weights for all newborns, infants, under five children and pregnant mothers in the community and health facilities by providing weighing scales (infant, adult).
- Malaria diagnosis using the rapid diagnostic tests in the community and health facilities
- Haemoglobin estimation using Haemocue in the community and at the facilities

#### Project goal

To contribute to improvement of maternal and child health in Kenya through health system strengthening initiatives at community and primary care levels.

#### Specific objectives

1. Conduct baseline survey on current levels of maternal and child health indicators, status of health system and existing programs in all the study areas.
2. Design and implement EHC package using the FLTR to improve access and quality of care of maternal and child health at tier 1 and 2 in one intervention cluster.
3. To explore and facilitate partnerships for innovative approaches (IGAs and Chamas) to incentivize CORPs (Community owned resource persons - volunteers), CHWs and Community Midwives (CMWs) to effectively participate in increasing access and retention of pregnant women and children in an intervention cluster.
4. To assess the effectiveness of the implementation of FLTR and EHC in the improving the efficiency of the referral system between the community (level 1), primary care facilities (level 2) and County referral facilities (level 3) for pregnant women and children in the intervention area.
5. Tobuild capacity of health workers to conduct health systems research and inculcate a culture of use of evidence to strengthen the performance of health systems through on-job training, in-service certificate and degree programs.
6. To conduct an endline survey and evaluations on levels of maternal and child health indicators, status of health systems and existing programs in the study areas at the end of the project in all the study clusters.

## Methods

We are implementing a quasi-experimental community trial. This design allows a comparison of outcomes between the intervention and control arms (Table 5). This quasi-experimental design entails:

- Implementation of the EHC through the FLTR strategy in one cluster (Obekai) in Busia.
- Facilitation of IGAs/CBOs in the implementation of the health care system in one cluster(Kabula) in Bungoma.
- Use of four control sites of Kiminini in Trans Nzoia,Chesunet in UasinGishu, Nyaporo in Kakamega and Nyaru in ElgeyoMarakwet county.
- We are implementing the project in a phased manner:

- ◯ Phase1: In year one we shall carry out a baseline survey in all the six clusters.
- ◯ Phase 2: We shall initiate interventions in two clusters (EHC/FLTR in Obekai and IGAs/CBOs in Kabula through community participation and ownership. These interventions will be implemented in year 2 toyear 4.
- ◯ Phase 3: Will consist of an endline survey and evaluation of the project in year 5.

### Setting

We shall utilise 6 of the 11 counties that Moi University frequently utilises for its health oriented outreach and extension activities. The six are Uasin Gishu, ElgeyoMarakwet, Trans Nzoia, Bungoma, Busia and Kakamega. The other counties used by the University not selected include Nandi, Baringo, West Pokot, Vihiga and Kisumu. One Dispensary was randomly selected from each of the six selected counties from the list in the e-Health Kenya platform.

### Study population

The study population will be all pregnant women living within the catchment population of the dispensary which is a location (Cluster) headed by a Chief, their newborns and under-five children born during the life of the project.

Two clusters will be randomly selected. One cluster will used as the intervention cluster for the implementation of the EHC through the FLTR strategy over a 3 year period.

The other will be used to implement the community owned and driven health care by encouraging the formation of income generating activities and community based organisations that will be used to fund the level 1 and level 2 health care services over the 3 year period of implementations.

The remaining four clusters and affiliated dispensaries shall be the control clusters in which only baseline and endline/evaluation data will be collected in years 1 and 5 respectively with no intervention implemented.

The heath centres, sub-county and county hospitals will be evaluated in the baseline and endline/evaluation phases of the study in all the 6 clusters.

Previous studies in these areas have shown an estimated 600 pregnant/newborn dyads per cluster per year. We shall therefore expect to enrol 1800 pregnant women/newborn dyads in each intervention cluster (EHC/FLTR intervention cluster and the Partnership using IGAs/CBOs cluster). The expected underfive year old population to be followed up during the study period will be about 5400 for each of the two intervention clusters. In the control clusters we expect to collect data for 6 months in each cluster and therefore obtain information for about 1200 pregnant women/newborns in all the three clusters at baseline and a similar number at endline.

### Inclusion criteria

- All pregnant women in the study clusters who give written consent.
- All newborn babies, infants and under five children born during the life of the study within the study cluster.

### Exclusion criteria

- Women not resident in the cluster during the study period.
- Newborns, infants and underfive children not resident in the study cluster.
- Infants and underfive children born before onset of the study in the study clusters
- Women living in the cluster that decline to give written consent

#### Study approach and analysis

##### 3.4.1.1.Baseline assessment

Conduct baseline survey on current levels of maternal and child health indicators, status of health system and existing programs in the study areas.

We aim to map the structure and functions of the primary health care system.

In Tier 1, this will be achieved by documenting the numbers, structures, functions and activities of the community units in the selected counties. Key stakeholders including among others, CHWs and community midwives will be identified as well as mechanisms for incentivizing them for their engagement.

At Tier 2, we will assess the functionality of each of the facility using the Structure, Process and Outcome approach as described in the Kenya Quality Model (KHSSP, 2013–2017).

In terms of Structure the specific variables of interest include among others: working hours, human resource, Number of staff and training according to the ministry of health standards, basic EMONC Equipment, supplies and commodity security.

For Processes we will assess availability of job aids such as protocols and guidelines

Lastly, in terms of Outcomes we will assess ANC indicators including gestation age at booking for ANC, Facility delivery, Signal functions for BEMONC. We will also assess the existence of nutritional, immunization and other IMCI services for under-five year old children.

At Tier 3, we will use the same approach as for Tier 2 to assess the same parameters in addition to existence of CEMONC signal functions.

Further in the baseline survey we will review the referral system from Tier 1 to Tier 3assess the strategies, referral destination, costs, types of cases referred and linkage success rate for all levels. Between Tier 2 and Tier 3, we will document the type of communication between the facilities and referral protocols and existence of ambulances.

We will assess the mechanisms used for data capture for the HMIS including, the tools, data collectors, where stored, reporting and how the data is consumed locally.

The baseline assessment will document information on challenges of motivating volunteers, existing and potential partnerships relevant to incentivizing CORPs and CHWs through CHUs. Appropriate information will be collected on willingness to initiate such partnerships and any past experiences relevant to their functioning and sustainability.

### Design and analysis

Baseline data collection forms will be developed and data collected and entered into a data base in the computer and analysed using the STATA version 12.

To assess maternal and child health indicators, status of health system and existing programs in the study areas, descriptive statistics will be used for the quantitative data while thematic areas will be derived from the qualitative data.

The results of the baseline survey will be disseminated to the stakeholders.

**Objective 2:** ***Intervention:*** Design and implement EHC package using the FLTR strategy to improve access and quality of care of maternal and child health at tier 1 and 2 in one intervention cluster.

The study population will be all pregnant women living within the catchment population of the dispensary whose catchment population is a location (Cluster) headed by a Chief, their newborns and underfive children born during the life of the project. The intervention will be implemented in this one cluster through the FLTR strategy over a 3 year period.

**Implementation of the intervention model** – We shall apply the FLTR (Find, Link, Treat, and Retain) strategy to support a successful implementation of Enhanced Health Care (EHC). This requires direct investment in health system elements that directly and indirectly contribute to efficiency of the referral system between the community and primary care levels and the County referral facilities under the new 4-tier health system.

**Find** – will involve investment in the Human Resources for Health (HRH) component of the health system by influencing recruitment of adequate and competent CHWs from within intervention villages and skilled health workers (SHWs) to follow up. It will involve investment in improvements in management components through strengthening of supervision by trained health workers. There will be use of appropriate technologies and supplies (computers, HMIS, mobile phone and household registers) to improve efficiency and strengthen the health information system.

**Link** - CHWs will encourage prompt ANC/MCH clinic attendance and schedule ANC/MCH clinic appointments by mobile phone device at the time of initial CHW visit (Link). This links directly to efficiency in Service delivery, investment in technologies (mobile phone) and health information for efficient management of referrals. This will create a link between level 1 and level 2 of the health system.

**Treat** - The ANC/MCH clinics in the facilities will provide the complete EHC package to each subject and will mainly address the quality of service delivery, technologies and commodities component. These include haemoglobin testing using the haemocue machine and malaria test using the Rapid Diagnostics Tests (RDTs). Any ailments will be treated at the local facility and where referral is needed they will be referred to the higher level facility. Newborns will be given the routine vaccinations, weights taken and prenatal and vitamin A provided including all the relevant services as outlined in the EHC package.

We shall be investing in improvement of quality of care through refresher training of all the skilled health workers in the study dispensary on MNCH, EmONC, ENC, customer care including public relations. The CHWs/CORPs will also be trained using the WHO guidelines on pregnancy surveillance and newborn follow up. This will include on methods of approach to household and villages and confidentiality. We shall provide essential equipment, drugs and dressings as per the essential commodities list of the Ministry of Health for the level 2 facility.

We shall utilise the modified MOH SOPs for this level of care for the management of illnesses at this level. We shall also provide for acceptable waiting time for patients as recommended and will conduct an exit survey for all mothers who are treated at this facility as they leave the facility on the same day.

**Retain** - Subjects will be tracked and if one defaults the CHW will be dispatched to the subject’s home to counsel the woman/family and accompany the woman to the health facility for the ANC/MCH visit. This component of the intervention links to building efficient and effective health information system that allows for efficiency in monitoring/tracking of subjects at antenatal, delivery and postnatal care. The component will also cover cost-reduction in tracking by applying appropriate technologies such as use of mobile phone. The follow up therefore will range from 6 months to 5 years depending on the month and year of birth over the 5 year study period. This translates into the retention of the women and children in the health system throughout the period of the study. This FLTR strategy will only apply to enrolled pregnant mothers and their infants within the intervention cluster. Women who become pregnant for the second or third time during the 5 year study period will be enrolled and followed up as per protocol. There will therefore be a possible 6 month to 5 year prospective follow up of the mother infant/child dyads once enrolled. There will therefore be cohorts of varying follow up periods ranging from 6 months and 5 years depending on when they are enrolled over the life of the study.

The following will be evaluated at baseline and at endline in the 1^st^and 5th year of the study:

- Increase in antenatal care attendance by pregnant women in the health facilities
- Increase in proportion and number of women delivering in health facilities
- Reduction in the rate of low birth weight and premature babies born
- Reduction in the rates of poor pregnancy outcomes including birth asphyxia, neonatal sepsis, neonatal mortality, maternal mortality, near miss maternal morbidity among pregnant women
- Exclusive breastfeeding rate, immunization completion rate, Infant and Young Children Feeding (IYCF) and IMCI parameters, anthropometric indicators.

**Objective 3:** ***Innovative partnerships:*** To explore and facilitate partnerships for innovative approaches (IGAs and Chamas) to incentivize CORPs (Community owned resource persons - volunteers), CHWs and Community Midwives (CMWs) to effectively participate in increasing access and retention of pregnant women and children in the health care system in the intervention cluster.

In this one cluster, we shall work with the community Health Units, village elders, Assistant chiefs and the chief in collaboration with the CHEWs and CHWs to establish income generating units (IGUs) and/or community based organizations (CBOs) in the cluster. The IGUs/CBO will be set up by the communities themselves and run through governance structures established by the communities themselves. The funds from these IGUs/CBOs will be used to incentivize the volunteer CHWs/CORPs to identify pregnant women and their newborns to the local dispensary. The primary care health facility committee will work with these CBOs and IGUs to assist in the funding of some activities including referral costs from the community to the primary care health facility and to the county referral facilities. These IGUs/CBOs will be used to address the transport and birth plans requirements of the pregnant women, their newborns and infants born during the life of the project.

The study population will be all pregnant women living within the catchment population of the dispensary whose catchment population is a location (Cluster) headed by a Chief, their newborns and underfive children born during the life of the project.

We will compare process and outcome indicators at baseline and at end of study for this cluster.

**Objective 4:** ***Efficiency of referral system:*** To assess the effectiveness and sustainability of the implementation of FLTR and EHC in the improvement of the efficiency of the referral system between the community (level 1), primary care facilities (level 2) and County referral facilities (level 3) for pregnant women in the intervention area.

The effectiveness and efficiency of the referral systems will be evaluated in the intervention clusters and compared at baseline to endline in the two clusters where interventions will be applied.

The number of referrals, timeliness of referrals, outcomes of referrals in terms of deaths and feedback provided to the referring facility by the referral facility, access of the referred patients to the doctors and consultants, transport availability and access, availability of referral notes and the detail of the referral and the feedback to the referring facility. Interaction between the referring and referral facility in terms of visits, calls, letters, e mails, frequency of supervisory visits etc. will be recorded and analysed.

**Objective 5: Capacity building:** To build capacity of health workers to conduct health systems research and inculcate a culture of use of evidence to strengthen the performance of health systems through on-job training, in-service certificate and degree programs.

This will be done through training of masters and doctoral students within the program and beyond in the health systems discipline. These masters and doctoral students will work with thehealth workers and community health workers during the study and implement their research projects at these sites. They will participate in the training and service provision while doing their research projects.

Skilled health workers in the two intervention clusters will also be trained on the job in various areas of relevance to health systems including health systems management, project management and health services management. The health workers will also be taken through refreshers courses on neonatal resuscitation, BEmONC, CEmONC, MNCH, maternal care during labour, breastfeeding, feeding for infants and older children, ENC. This is with the aim of ensuring they provide quality care. They will be standardised every six months to ensure their performance is consistent throughout the study period.

### Objective 6

Conduct endline survey/evaluation on levels of maternal and child health indicators, status of health system and existing programs in the study areas at the end of the project in all the study clusters.Theendline survey and evaluation will be carried out in the 6 clusters that were included in the study.As in the baseline survey, we shall document the numbers, structures, functions and activities of the community units in the study counties in the fifth year of the study and the same data forms and tools used for the baseline survey will be utilised in tiers 1 and 2.

In terms of Structure the specific variables of interest include among others: working hours, human resource, number of staff and training according to the ministry of health standards, basic EMONC Equipment, supplies and commodity security.

For Processes we will be interested in availability of job aids such as protocols and guidelines.

Lastly, in terms of Outcomes we will be interested in ANC indicators including gestation age at booking for ANC, Facility delivery, Signal functions for BEMONC. We will also assess the existence of nutritional, immunization and other IMCI services for under 5 year old children.

At Tier 3, we will use the same approach as for Tier 2 to assess the same parameters in addition to existence of CEMONC signal functions.

As in the baseline survey we will be interested in the referral system from Tier 1 to Tier 3. For all the levels we will be interested in the strategies, referral destination, costs, types of cases referred and linkage success rate. Between Tier 2 and Tier 3, we will also be concerned on the type of communication between the facilities and referral protocols and existence of ambulances. We will also assess the mechanisms used for data capture for the HMIS including, the tools, data collectors, where stored, reporting and how the data is consumed locally.

The baseline data collection instruments will be used for the endline survey using the same methodology, data analysis and dissemination.

#### Anticipated outputs and outcomes

Table 1 shows the expected deliverables, their link to outcomes and how these will influence policy among the beneficiaries.

**Table 1:** Anticipated outputs and outcomes and their likely policy influence

Major research outputs/ products	Expected outcomes	Likely policy influence – outcome challenges on targeted audience
Reports (3)	Mapping of gaps from baseline indicators and benchmarking for priority interventions in various contexts EHC through FLTR strategy contextualized (if necessary) Case studies and best practices for the intervention defined	Community/beneficiaries sensitized for ownership and effective participation in programme Stakeholders sensitized on benchmarks and priority interventions from baseline findings Policy makers sensitized innovations around implementation of the EHC / FLTR strategy Evaluation findings packaged to reveal best practices for possible scale up to other areas
Phase 1: baseline report		
Phase II: Intervention report		
Phase III: Evaluation report		
Policy briefs (4)	Documentation on how implementation of EHC through the FLTR strategy (1st arm of the intervention) and innovations through CORPs (2nd arm of the intervention) lead to improvements access to and quality of care by pregnant women, neonates and infants reductions in morbidity among pregnant women, neonates and infants referral services for MNNH at county level (tier 1 to 3)	It is anticipated that the three briefs aimed at policy makers should demonstrate *How investments in EHC through the FLTR strategy triggers health system changes that lead to improvements in access and quality of care for pregnant women, newborn and infants. *How innovations in resourcing CORPs can incentivize CHWs, CHCs and CHEWs, enhance access via innovative transport arrangements through sustainable birth preparedness plans at tier 1 of the health system *How application of ICT / mobile health at tier 1 to improve quality of care through efficient clinical decision support systems that are cadre specific and aligned to MOH DHIS tools *CORPs used as birth companions leading to timely and appropriate referrals that minimize maternal and newborn morbidity and mortality between tier 1 and 3
Abstracts and posters (12) presented at Moi University annual scientific conference and other conferences by project staff (6) and post graduate students (6)	Demonstrated effect of interventions on intermediate (access and quality of care) as well as long-term outcomes (Maternal, neonatal and infant health indicators) as per study objectives	The abstracts will communicate to national and international audience of policy makers, planners and implementers on best practices and share lessons as per programme objectives
Peer reviewed Journal articles(7)	Several publications from study: 1. Document situation analysis (at baseline) on maternal, neonatal and child health 2. How FLTR strategy and EHC lead to improvements in access 3. How FLTR strategy and EHC improve quality of care 4. How interventions lead to improvements in maternal, neonatal and infant health 5. How innovations incentivize CORPs and lead to their sustainable engagement 6. How integrating eHealth using mobile phone technology improves HMIS and quality of care 7. systematic reviews from masters and doctoral students	Publishing peer reviewed articles will help policy makers articulate quality of evidence from the study and lead to evidence-based planning, policy making and practice Help raise the profile and role of University as partner in health towards achieving the Vision 2030
Thesis at masters level (6) Dissertations at doctoral (2)	Raise the knowledge base and skills of students and health workers in health systems research (HSR) Encourage placement and internships with policy making institutions (public and private)	Policy makers to invest in health systems research and support university chair in future HSR initiatives Improve ranking and image of University as a hub/centre for excellence in HSR
3 Guidelines / SOPs on EHC using the FLTR strategy	Efficiency of County referral services for MNH and referral strategy	New guidelines will help implementers improve performance and policy makers decide on approaches to scaling up intervention after the pilot phase will benefit from improvements
3 Curricula: New doctoral level (HSM) – 2 students Revised MPH (HSM) – 6 students Revised short course (HSR) – 10 students	Enhanced competencies – knowledge, skills and attitudes in HSR through the short course, MPH in HSM, and Doctoral programme in HSM Competent and performing graduates placed appropriately in the health system	Policy makers will lobby for and support scholarships in HSR relevant training – Implementers will be motivated to come and study – certificate, masters, doctoral level Document best practices in curriculum for HSR - core competencies and share with network of academic institutions implementing HSR relevant curriculum
Advocacy materials include: • pamphlets on programme interventions & practices – (produced bi-annually); • project web page to link to Moi University website updated regularly; • Press releases – TV and print media • Talks through bazaars etc.	Effective communication of stakeholders in the project planning, implementation and evaluation; Effective and sustained community engagement through the talks and other press releases; Enhances project governance - transparency & accountability to stakeholders	Policy makers can monitor and engage in analysis of policy such as Community health strategy Share programme knowledge with communities enhancing the learning curve from best practices and lessons for scale up of interventions in similar or other contexts

#### Dissemination plan

The dissemination plan is shown in Table 2 to ensure the right information is shared with the right stakeholders at the right time.

**Table 2:** Dissemination plan of key messages to various target audiences

Target audience	Key message	Proposed channels of communication
NACOSTI	All matters of the project as specified in the agreement	Seminars, meetings, reports, financial and technical reports; registration of patents and innovations
Policy makers and decision makers	Evidence that informs rationale for policy change, need for reforms; innovations; cost-effective interventions; updates on specific project portfolios	Policy briefs, stakeholder forums, website, dialogue days, newsletters
Project staff and sponsors and project stakeholders	project organization and governance structure; vision, mission, objectives, scope of project activities; legal provisions (agreements signed); stakeholders and roles; progress on implementation; project products;	Project website; newsletter, brochures, public meetings, press/media briefs; twitter or other appropriate feedback from the public; dialogue days
Media	Messages on project progress/ updates; announce conferences, seminars, innovations; advocacy messages will depend on target audience;	Print and audio channels; press/briefs; seminars national conference
Advocacy groups and civil society	Messages that require dialogue with various stakeholders	Public forum, media, website, twitter and other appropriate social media to get feedback
Students	curriculum, recruitment and implementation, fee structure, requirements for various programs; evaluation and feedback; upcoming seminars, student exchanges, other collaborations, seminar opportunities, funding for HSR activities; awards etc	Website, approved senate curriculum, official advertisements on enrolment and graduation lists; student discussion forums on the websites; class schedules, formal examination, transcripts and certificates; thesis and published papers in journals
Regulatory bodies such as Commission for University Education, Medical Practitioners and Dentist Board, Nursing Council, Pharmacy and Poison Board, Lab; Legal	Establishment and monitoring of standards and norms; guidelines for various programmes on certification and licensing; legal procedures and MoUs/MOAs	Site visits, share SOPs reports; documents; meetings; certificates of approval; registration of patents and innovations on the project
Mobile phone provider	Guidelines, SOPs, information required, source; timing and target stakeholders	phone, SMS, discussion forums, alerts, help lines (hot lines); other services
Community/ public	Relevance of project priorities; role in project implementation; project products; co-funding where appropriate; issues that require consensus; right to representation on project committees; accountability and transparency of project /governance structures	Public forums; brochures; meetings, membership to committees; progress on implementation; project products; seminars to train selected members on committees; minutes of key committees and reports on progress;

### Dissemination targets from the key objectives to cluster populations

***Objective 1:*** Conduct baseline survey on current levels of maternal and child health indicators, status of health system and existing programs in the study areas.

- Results of the baseline and endline surveys for all clusters will be provided to the respective counties (Table 3).

**Table 3:** Intervention and control clusters

	Name	County	Sub-County	Division	Type	Subcounty referral facility	County referral facility
1	Obekai (FLTR/EHC)	Busia	Teso South	Chakol	Dispensary	Nambalesubcounty hospital	Busia county hospital
2	Kabula (IGAs/CBOs)	Bungoma	Bungoma South	Bumula	Dispensary	Bungoma county hospital	Bungoma County Hospital
Health facilities for Health systems control arm							
	Name of dispensary	County	Sub-county	Division	Type	Owner	
1	Nyaporo	Kakamega	Mumias	East Wanga	Dispensary	MoH, Kakamega County	
2	Kesses	UasinGishu	Eldoret South	Kesses	Dispensary	MoH, UasinGishu County	
3	Matunda	Trans Nzoia	Trans Nzoia West	Kiminini	Dispensary	MoH, Trans Nzoia County	
4	Nyaru	Keiyo Marakwet	Keiyo South	Chepkorio	Dispensary	MoH Keiyo Marakwet County	

***Objective 2*** **:** Design and implement EHC package using the FLTR to improve access and quality of care of maternal and child health at tier 1 and 2 in one intervention cluster.

- Results of the baseline and endline surveys in the intervention cluster
- Health facility outputs and outcomes
- Exit survey results

**Objective 3:** To explore and facilitate partnerships for innovative approaches (IGAs and Chama) to incentivize CORPs (Community owned resource persons - volunteers), CHWs and Community Midwives (CMWs) to effectively participate in increasing access and retention of pregnant women and children in the one intervention cluster.

- Results of the baseline and endline surveys in the one cluster that this will be implemented
- IGAs/CBOs established and sustained and income generated
- Proportion of motivated volunteers and members of CHCs
- Improved performance targets amongst volunteers comparing intervention and control clusters

**Objective 4:** To assess the effectiveness and sustainability of the implementation of FLTR and EHC in the improvement of the efficiency of the referral system between the community (level 1), primary care facilities (level 2) and County referral facilities (level 3) for pregnant women in the intervention area

- Increase in the referrals to county facilities
- Proportional increase in referrals
- Pregnancy outcomes for women referred over study period compared to baseline

**Objective 5:** To build capacity of health workers to conduct health systems research and inculcate a culture of use of evidence to strengthen the performance of health systems through on-job training, in-service certificate and degree programs.

- Number of trained health workers, CHWs, CHU officials, County health officers
- Number of degree graduates trained
- Number of certificate graduates
- Number of guidelines developed

***Objective 6:*** Conduct end line survey on levels of maternal and child health indicators, status of health system and existing programs in the study areas?

- Results of the endline surveys for all clusters
- Study outputs and outcomes
- Lessons learnt
- Areas for improvement
- Areas for further research and study.

#### Projected and possible levels of achievements at end of the study

The possible levels of improvements in the indicators is shown in Table 4 as low, medium and high with low meaning modest improvement in indicators and high meaning significant improvement.

**Table 4:** Projected/expected Levels of project outcome/improvement

Starting point (baseline)	Low outcome level	Moderate outcome level	High outcome level
Average figures from KDHS which will be confirmed at baseline)			
Antenatal care attendance by pregnant women in the health facilities (currently about 46%)	50	60	85
%of pregnant women completing 4 ANC visits (45%)	50	70	90
Reduce the rate of low birth weight and premature babies born in the study population (LBW currently is about 4.1%)	4.1	3.8	1.8
Reduce the rates of poor pregnancy among pregnant women in the study population from			
birth asphyxia from 25%,	23.5	17.5	10
neonatal sepsis - 25%,	23.5	17.5	10
neonatal mortality −35%,	32.5	24.5	15
maternal mortality – 488/100,000,	400	350	250
Proportion of skilled health workers trained to provide quality health care (%)	8	10	15
Proportion of women satisfied with quality of services provided at primary care facilities (%)	35	55	80
Increase the number of health personnel with competencies participating in health systems research and using evidence to make decisions			
• certificate	5	10	30
• masters	3	6	10
• doctoral	1	2	3
Improve efficiency of the referral system between the community (tier 1), primary care facilities (tier 2) and County referral facilities (tier 3) for pregnant women in the intervention area (currently unknown)	Qualitative		
	Accountability, transparent, participation in decision making, client satisfaction		
Sustained engagement of motivated CHWs, CMws making timely referrals of pregnant women and mothers with under 5 years (%)	30	50	80
Proportion of timely referrals between tier 1 & 2			
Proportion of established IGUs/CBOs are functional Nature of governance structures and processes, leadership and management practices as well as viability/sustainability of the established IGUs/CBOs post URCP programme	30	60	80

#### Monitoring and evaluation strategy

Table 5 shows what will be monitored and how it will be done including the evaluation. The contribution of the project includes improvement in quality of maternal and child health care, reduction in Maternal Mortality ratio, increase in skilled birth attendance at delivery, reduction in stunting among under-fives, reduction in the under five mortality rate, reduction in Infant mortality rate, increase in fully immunized infants at <12 months, and the number of masters and doctoral graduates.

**Table 5:** Programme monitoring and evaluation framework

Goal:								
To contribute to improvement of maternal and child health in Kenya through health system strengthening initiatives at community and primary care levels								
Objective 1:Conduct a baseline survey on maternal and child health interventions to improve access and quality of care at tiers 1 and 2 in select counties								
Activities/inputs	Output/Deliverables	Means of verification	Objectively Verifiable Indicators	Timelines				
				2015	2016	2017	2018	2019
Review and develop tools	Reviewed tools Develop tools	Survey tools	# tools					
Recruit survey team	Survey team in place	Advertisements, Interviews	letters of appointments					
Train survey team and pilot tools	Trained RAs	Logistics Venue	# trainings, # staffs trained					
Prepare for Community entry	Logistics and costs Set dates for entry	Sensitized community	# meetings with the community and county					
Conduct the survey	Survey instruments	Baseline data	# field tools					
Develop data entry template	Software	Ready template	Templates for qualitative & Quantitative data					
Data entry and interpretation	Data clerks Software	Entered data	Data in template					
Analyze and write report	Draft report	Findings/Report	# Reports					
Disseminate the findings	Write ups	Reports	# dissemination meetings # media appearances # policy briefs					
Objective 2:Adapt and implement the EHC package using the FLTR strategy to improve access and quality of care of maternal and child health at level 1 (community) and level 2 (primary care facilities) in the intervention cluster,								
1. Identification CORPs-Domiciliary nurses and CHVs in specified counties	Write ups	MOUs Contract letters	#CORPs					
2. Training	Curriculum	Pre & post assessment	#trainings #staffs trained					
3. Review HMIS forms	Forms	Types of forms	#forms reviewed					
4. Prepare the community for intervention		Minutes/ reports	#sensitization meetings Budgets					
5. Roll out the intervention	Intervention in place	HF records, endline evaluation	changes key indicators					
Objective 3: To explore and facilitate partnerships for innovative approaches (IGAs and Chama) to incentivize CORPs (Community owned resource persons - volunteers), CHWs and Community Midwives (CMWs) to effectively participate in increasing access and retention of pregnant women and children in the intervention cluster.								
6. Review documents and identify potential partners	Functional CBOs, Chamas, SACCO	Agreements/ MOUs	#partners #MOUs #Meetings with partners					
6.Develop advocacy communication and social mobilization strategy for the programme	sensitized community, policy makers, healthworkers	Meetings Minutes Reports Documents	#meetings #media appearances #ACSM strategy					
7. Create partnerships	private sector engaged	Minutes MOUs	#CORPs engaged #Task shifting- CORPs as birth a					
8. Review documents and identify potential partners	sustainable partnerships	Agreements/MOUs	#partners #MOUs #Meetings with partners					
Objective 4: To assess the effectiveness and sustainability of the implementation of FLTR and EHC in the improvement of the efficiency of the referral system between the community (level 1), primary care facilities (level 2) and County referral facilities (level 3) for pregnant women in the intervention area								
Activities	Output	Means of verification	Objectively Verifiable Indicators	Timelines				
				2015	2016	2017	2018	2019
Develop advocacy communication and social mobilization strategy for the programme	sensitized stakeholders	Meetings Minutes Reports Documents	#meetings #media appearances #ACSM strategy					
Provide Mobile telephony to CORPs	quality data collected & used	Numbers Timely referrals Usage	#mobile phones provided					
Conduct a customer satisfaction survey and use lessons learnt to improve service	satisfied pregnant women & mothers	Report Survey Tools	Report #recommendations Improvement in quality					
Determine numbers of still births	reduced no. still births	Numbers	#still births					
Objective 5: To build capacity of health workers to conduct health systems research and inculcate a culture of use of evidence to strengthen the performance of health systems through on-job training, in-service certificate and degree programmes								
Recruit program staff and monitor their performance	SHWs	Appointment letters Reports	#staffs recruited Improved service					
Develop short courses and enhance capacities in leadership, governance and management of systems	HSR, LMG skills	Curriculum Trainings	#Curriculum #Trainings					
Admit 4 masters’ students into the program and assist them choose topics relevant to the program, determine gaps and propose interventions	graduates, thesis and publications	Graduates Titles of study	#graduate students admitted Progression rates #intervention gaps Policy influence Abstracts Manuscripts Conference appearances					
Admit 2 PhD candidates within the 5 year period and involve them in identifying research problem and designing interventions	admissions graduates thesis articles	Candidates Titles of study	#candidates admitted Progression rates #intervention gaps Policy influence Abstracts Manuscripts Conference appearances					
Sensitize CORPs on safe motherhood	sensitizes CORPs	Sensitization sessions Numbers Reports	#trainings Change in knowledge & attitudes					
Training on indications for referral of pregnant mothers and sick child	KSP for CHWs & CORPs	enhanced Curriculum	#CORPs trained #health talks					
Objective 6:Conductendline survey/evaluation on levels of maternal and child health indicators, status of health system and existing programs in the study areas at the end of the project in all the study clusters.								
Review baseline tools to include indicators on program relevance and effectiveness	revised tools	hard copies	# of tools					
Recruit and train RAs	trained RAs, tools	survey teams	minutes, letters, list					
Collect data	completed tools	field schedule	letters of release and payment schedule					
Analyze and interpret data	tables, results	hard copies	outputs and verification forms					
Write evaluation report documenting findings; project report (overall)	reports	draft reports	submission of drafts, feedback, meeting with stakeholders					
Disseminate findings to appropriate audience and partners	report briefs	minutes, emails, feedback	letters/emails submission, final feedback					

#### Ethical considerations

The proposal will be approved by the Institutional Research and Ethics Committee of the Moi University and by the 6 County Health Management Teams (CHMTs).

### Consent process

The written consent will be obtained from all women enrolled into the study who accept to participate. Those mothers who will be minors (age 12 years and above) will have their consents provided by their guardians or husbands. Illiterate eligible women will have their thumb prints taken and witnessed by an independent adult. The IREC guidelines on confidentiality and on research among vulnerable groups will be followed.

#### Declaration

*“This work was carried out with the financial support from the National Commission for Science, Technology and Innovation (NACOSTI) and the International Development Research Centre (IDRC) Canada. The views expressed in this work are those of the creators and do not necessarily represent those of the National Commission for Science, Technology and Innovation, and the International Development Research Centre, Canada or their Board of Governors.”*

#### Project timelines

The project timelines are shown on Table 6

**Table 6:** Proposed project timeline

Project activities	Year 1	Year 2	Year 3	Year 4	Year 5
*Planning meetings with project partners*					
*IREC Proposal approval*					
*Implementation planning meetings by study team*					
*Implementation planning meeting with stakeholders (MOH, County Health teams, NACOSTI, IDRC etc)*					
*Community entry*					
*Piloting study instruments*					
*Start of study proper – intervention*					
*Monitoring*					
*Data collection control clusters*					
*Data collection intervention clusters*					
*Data entry*					
*Data cleaning*					
*Data analysis*					
*Training of masters and doctoral degree students*					
*Training of CORPs & CHEWs*					
*Final report writing and study closure*					

## Discussion

Most African countries did not meet the MDGs on reducing child mortality, improving maternal health and combating infectious disease (MDGs4, 5 and 6). Yet, experiences from other continents, as well as recent progress in several countries in the region, prove that the goals can be achieved across Africa. Nevertheless, support for rapid scale-up of proven interventions as well as critically needed investments in basic healthcare systems remains insufficient [24]. In most African countries the basic health infrastructure, human resources, equipment and supplies are inadequate to provide essential maternal, child and reproductive health services, and to control and treat infectious diseases. Malaria and other vector-borne diseases that can be controlled and treated continue to take millions of lives throughout Africa and are spreading to more parts of the continent (MDG Report by African Steering group, [23]).

The relevance of the health system to maternal and child health can be systematically captured through the building blocks [1]. An efficient and effective health care system requires a leadership and governance structure, a health care financing system, human resources, a health care delivery system, drugs and equipment and a health information system that is robust and accessible.

*Leadership and Governance* have been observed the world over as an important need for the success of any health care system. This is a requirement for the success of the health care delivery system at all level of care (level 1 to 4 in Kenya).

*Healthcare financing* is a major hindrance to adequate and satisfactory health care delivery in any country and has been cited as one of the major challenges in developing countries in health care delivery.

*Service delivery* is a complex sub-system for both developed and developing countries. A functional system should aim to provide safe and quality, feasible, affordable and accessible healthcare. Many countries have tried several health service delivery models and hospital reforms without much success and even the most developed countries are still grappling with achieving optimal health care systems. This requires a systematic approach to collect, interpret and use evidence to inform and improve practices within the health system.

*Medical products, vaccines and technologies* are a necessity in a health care system without which utilization slows or is poor and therefore must be procured and made available in health facilities.

*Health Infrastructure is a crucial sub-system as m*ost health systems in Sub-Saharan Africa are dilapidated and new infrastructure is poorly maintained. A well-functioning health system ensures equitable access to healthcare by ensuring physical infrastructure is available within the required distance, transport infrastructure ensures speedy access and referrals and ICT infrastructure supports timely procurement of supplies and equipment and facilitates effective management of resources.

*Health management information system (HMIS)* is key in a health care delivery system and has to be functional for a successful system of health care. Mobile phone technology and a Medical Records System are important in a health delivery system in order to obtain disease surveillance, demographic and statistical data for planning and follow up

**Fig. 4:**
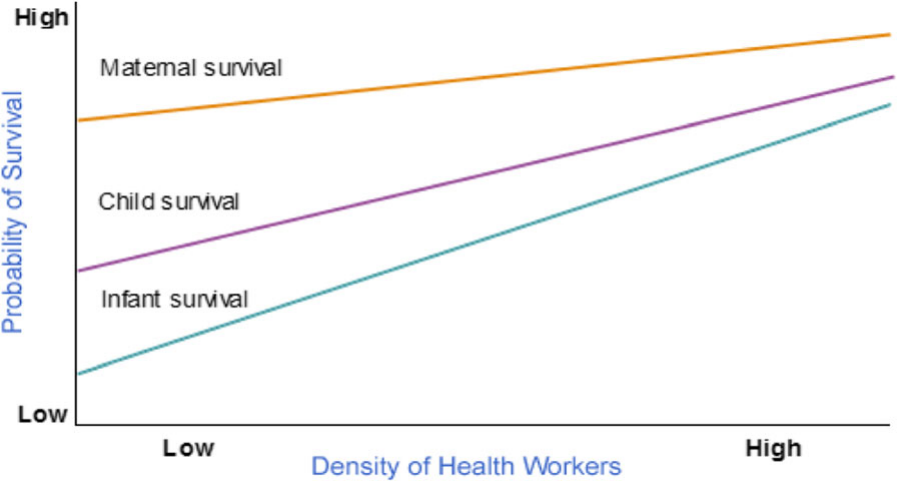
Relationship between Human resource density and maternal and child survival

*Human resources* are an essential component of a health system and a major shortcoming in sub-Saharan Africa where a deficit of over one million health workers is estimated to exist and Kenya is no exception (WHO 2006). There is a direct linear relationship between the density of human resources in health to maternal and child survival as shown in Fig. 4. In Kenya the health human resources to population ratio is 13/10,000 populations compared with the WHO figure of 23/10,000 population threshold [23, 25].

This project intends to utilize these pillars of the health system to address most of the challenges encountered in health care delivery in Kenya and other low middle income countries over the last several decades.

## Conclusions

The results of this study will be useful to policy makers in Kenya and other developing countries in the formulation of guidelines and policies during the revision of their existing health systems in the national health service especially at the primary care level.

## Abbreviations

ANC: Antenatal care
BEMONC: Basic Emergency Obstetric and Newborn care
CBO: Community Based organisations
CEMONC: Comprehensive emergency Obstetric and Newborn care
CHEWs: Community Health Extension Workers
CHUs: Community Health Units
CHWs: Community Health Workers
CMWs: Community Midwives
CORPs: Community owned resource persons
EHC: Enhance health care
EMONC: Emergency Obstetric and newborn care
ENC: Essential newborn care
FLTR: Find Link Treat Retain
HSR: Health systems research
IGUs: Income generating Units
IMCI: Integrated Management of Childhood Illnesses
KDHS: Kenya demographic health survey
LLITNs: Long lasting insecticide treated nets
MCH: Maternal child health
MDGs: Millenium development goals
MNCH: Maternal Newborn Child health
SDGs: Strategic development goals
SHWs: Skilled health workers
UN: United Nations
UNICEF: United Nations Childrens education fund
WHO: World Health organisation
